# EGCG, a major component of green tea, inhibits tumour growth by inhibiting VEGF induction in human colon carcinoma cells

**DOI:** 10.1054/bjoc.2000.1691

**Published:** 2001-03

**Authors:** Y D Jung, M S Kim, B A Shin, K O Chay, B W Ahn, W Liu, C D Bucana, G E Gallick, L M Ellis

**Affiliations:** Chonnam University Research Institute of Medical Sciences, Chonnam University Medical School, Kwangju, 501-190;, Korea; Departments of Cancer Biology and; Departments of Surgical Oncology, The University of Texas MD Anderson Cancer Center, Houston, Texas, 77030, USA

**Keywords:** epigallocatechin gallate (EGCG), vascular endothelial growth factor (VEGF), colon carcinoma, Erk-1, Erk-2, angiogenesis

## Abstract

Catechins are key components of teas that have antiproliferative properties. We investigated the effects of green tea catechins on intracellular signalling and VEGF induction in vitro in serum-deprived HT29 human colon cancer cells and in vivo on the growth of HT29 cells in nude mice. In the in vitro studies, (-)-epigallocatechin gallate (EGCG), the most abundant catechin in green tea extract, inhibited Erk-1 and Erk-2 activation in a dose-dependent manner. However, other tea catechins such as (-)-epigallocatechin (EGC), (-)-epicatechin gallate (ECG), and (-)-epicatechin (EC) did not affect Erk-1 or 2 activation at a concentration of 30 μM. EGCG also inhibited the increase of VEGF expression and promoter activity induced by serum starvation. In the in vivo studies, athymic BALB/c nude mice were inoculated subcutaneously with HT29 cells and treated with daily intraperitoneal injections of EC (negative control) or EGCG at 1.5 mg day^−1^mouse^−1^starting 2 days after tumour cell inoculation. Treatment with EGCG inhibited tumour growth (58%), microvessel density (30%), and tumour cell proliferation (27%) and increased tumour cell apoptosis (1.9-fold) and endothelial cell apoptosis (3-fold) relative to the control condition (*P*< 0.05 for all comparisons). EGCG may exert at least part of its anticancer effect by inhibiting angiogenesis through blocking the induction of VEGF. © 2001 Cancer Research Campaign http://www.bjcancer.com


*British Journal of Cancer* (2001) **84**(6), 844–850
© 2001 Cancer Research Campaign
doi: 10.1054/ bjoc.2000.1691, available online at http://www.idealibrary.com on IDEAL®

### EGCG, a major component of green tea, inhibits tumour growth by inhibiting VEGF induction in human colon carcinoma cells

**YD Jung[1,2], MS Kim[1], BA Shin[1], KO Chay[1], BW Ahn[1], W Liu[2], CD Bucana[2], GE Gallick[2] and LM Ellis[2,3]**

[1]Chonnam University Research Institute of Medical Sciences, Chonnam University Medical School, Kwangju, Korea 501-190; Departments of [2]Cancer Biology and [3]Surgical Oncology, The University of Texas MD Anderson Cancer Center, Houston, Texas 77030, USA

**Summary** Catechins are key components of teas that have antiproliferative properties. We investigated the effects of green tea catechins on intracellular signalling and VEGF induction in vitro in serum-deprived HT29 human colon cancer cells and in vivo on the growth of HT29 cells in nude mice. In the in vitro studies, (-)-epigallocatechin gallate (EGCG), the most abundant catechin in green tea extract, inhibited Erk-1 and Erk-2 activation in a dose-dependent manner. However, other tea catechins such as (-)-epigallocatechin (EGC), (-)-epicatechin gallate (ECG), and (-)-epicatechin (EC) did not affect Erk-1 or 2 activation at a concentration of 30 μM. EGCG also inhibited the increase of VEGF expression and promoter activity induced by serum starvation. In the in vivo studies, athymic BALB/c nude mice were inoculated subcutaneously with HT29 cells and treated with daily intraperitoneal injections of EC (negative control) or EGCG at 1.5 mg $day^{-1}$ $mouse^{-1}$ starting 2 days after tumour cell inoculation. Treatment with EGCG inhibited tumour growth (58%), microvessel density (30%), and tumour cell proliferation (27%) and increased tumour cell apoptosis (1.9-fold) and endothelial cell apoptosis (3-fold) relative to the control condition ($P < 0.05$ for all comparisons). EGCG may exert at least part of its anticancer effect by inhibiting angiogenesis through blocking the induction of VEGF. © 2001 Cancer Research Campaign http://www. bjcancer.com

**Keywords**: epigallocatechin gallate (EGCG); vascular endothelial growth factor (VEGF); colon carcinoma; Erk-1; Erk-2; angiogenesis

Epidemiologic studies have shown that the consumption of green tea lowers the risk of developing gastric and colon cancers (Yu et al, 1995; Ji et al, 1997). In rodent models, green tea preparations protect against the development of skin, lung, mammary gland and gastrointestinal tract cancers (Rogers et al, 1998). The anticarcinogenic and antiproliferative effects of green tea have been attributed to the biological activities of its polyphenol components. Green tea extract contains (-)-epigallocatechin gallate (EGCG), (-)-epigallocatechin (EGC), (-)-epicatechin gallate (ECG), and (-)-epicatechin (EC) (Stoner and Mukhtar, 1995). EGCG, the most abundant polyphenol in green tea, has been shown to inhibit cell proliferation (Asano et al, 1997) and induce apoptosis (Hibasami et al, 1998; Paschka et al, 1998) in tumour cells. Other means by which EGCG may prevent cancer include its inhibition of urokinase activity (Jankun et al, 1997), mitogen-activated protein kinases (MAPKs) activation (Jankun et al, 1997; Ahn et al, 1999), lipooxygenase and cyclooxygenase activities (Stoner and Mukhtar, 1995), and arrest of the cell cycle (Ahmad et al, 1997; Fujiki et al, 1998) in tumour cells.

Signalling pathways that mediate proliferation also mediate other processes involved in tumour progression (Ellis et al, 1998; Jung et al, 1999). Tumour growth induced by mitogenic compounds is associated with the activation of several cytosolic proteins, including those involved in the phosphorylation and activation of MAPKs (Cobb and Goldsmith, 1995). Among the three subgroups of MAPKs in mammalian cells, the Erks seem to be the most important for growth factor-induced cell proliferation (Davis, 1993). Regulators of angiogenesis are also important determinants of tumour growth (Folkman, 1995) and various signal transduction pathways have been implicated in regulating angiogenic factor expression. Vascular endothelial growth factor (VEGF) is the angiogenic factor most closely associated with inducing and maintaining the neovasculature in human colon cancer (Takahashi et al, 1995, 1997; Ellis et al, 1996). Activation of Erk-1 and Erk-2 have been shown to be important mediators for up-regulation of VEGF mRNA. Milanini et al (1998) demonstrated that activation of Erk-1 and Erk-2 play a key role in the regulation of the VEGF expression via alteration of AP-2 and Sp1 transcription factors in fibroblasts. Our previous studies on VEGF induction in serum-starved cells revealed a causal role for the activation of Erk – but not of P38, stress-activated protein kinase (SAPK) or Akt (Jung et al, 1999).

A recent finding that green tea and one of its components, EGCG, prevents the growth of new blood vessels in animals (Cao and Cao, 1999) suggests a mechanistic link between the consumption of tea and the possible prevention and treatment of angiogenesis-dependent diseases, including cancer. In this study, we investigated the effects of green tea catechins on VEGF expression in vitro and their effects on tumour growth in vivo in human colon cancer xenografts in nude mice.

*Received 10 July 2000*
*Revised 8 November 2000*
*Accepted 17 November 2000*

*Correspondence to:* LM Ellis

### MATERIALS AND METHODS

### Reagents

Purified EGCG, EGC, ECG, and EC were obtained from Sigma Chemical Co (St Louis). Other reagents were obtained as follows:

optimum cutting temperature (OCT) compound from Miles Inc (Elkhart), diaminobenzidine substrate (DAB) and Universal Mount from Research Genetics (Huntsville), Superfrost slides from Fisher Scientific Co (Houston), terminal deoxynucleotidyl transferase (TdT)-mediated dUTP nick-end labelling (TUNEL) kit from Promega (Madison), and 4,6-diamidino-2-phenylindole dihydrochloride (DAPI) mount from Vector Laboratories Inc (Burlingame).

Antibodies for the immunohistochemical analyses were obtained as follows: rat anti-mouse CD31/PECAM-1 antibody from Pharmingen (San Diego); mouse anti-PCNA clone PC 10 DAKO A/S from Dako Corp (Carpinteria); peroxidase-conjugated goat anti-rat immunoglobulin (IgG) (H+L) and Texas Red- and fluorescein-conjugated goat anti-rat IgG from Jackson Research Laboratories (West Grove); and peroxidase-conjugated rat anti-mouse IgG2a from Serotec Harlan Bioproducts for Science Inc (Indianapolis).

### Cell culture

The human colon cancer cell line HT29 was obtained from the American Type Culture Collection (Manassas) and cultured in minimal essential medium (MEM) supplemented with 10% fetal bovine serum (FBS), 2 U $ml^{-1}$ penicillin and streptomycin, 1 mM sodium pyruvate, 2 mM L-glutamine, vitamins, and nonessential amino acids at 37˚C under 5% $CO_2$. Serum deprivation was induced by excluding FBS from the standard culture medium after cells had reached 90–100% confluence.

### Western blot hybridization

Cells were rinsed twice with ice-cold phosphate-buffered saline (PBS) and then lysed with protein lysis buffer (20 mM Na-phosphate (pH 7.4), 150 mM NaCl, 1% Triton X-100, 5 mM EDTA, 5 mM phenylmethylsulfonyl fluoride, 1% aprotinin, 1 μg $ml^{-1}$ leupeptin, and 500 μM $Na_3VO_4$). Protein levels were quantified spectrophotometrically, and 100-μg portions were subjected to electrophoresis on 8% polyacrylamide gels, transferred to nitrocellulose membranes (Schleicher & Schuell, Keene) by electrotransfer, blocked with 5% milk in PBS-T (0.5% Tween 20 in PBS), and probed with the primary antibody (1:1000 dilution of rabbit anti-phosphospecific p44/42 MAPK antiserum for phosphorylated Erk-1 and -2 (New England Biolabs Inc, Beverly).

The membranes were then washed and treated with secondary antibody labelled with horseradish peroxidase (anti-rabbit immunoglobulin antiserum from donkey at a 1:3000 dilution, Amersham, Arlington Heights). Protein bands were visualized with a commercially available chemoluminescence kit (Amersham). To quantify total Erk-1 and -2 protein levels, the membrane was washed with stripping solution (100 mM 2-mercaptoethanol, 2% SDS, and 62.5 mM Tris-HCl (pH 6.7)) for 30 min at 50˚C and reprobed with rabbit anti-p44/42 MAPK antiserum (New England Biolabs) at a 1:1000 dilution.

### mRNA extraction and Northern blot analysis

Total RNA was extracted from cells by using the Tri Reagent (Molecular Research Center Inc, Cincinnati). Northern blot hybridization was performed as previously described (Jung et al, 1999). In brief, total RNA (25 μg) was subjected to electrophoresis on 1% denaturing formaldehyde-agarose gels, transferred to a Hybond-$N^+$ positively charged nylon membrane (Amersham) overnight by capillary elution, and ultraviolet cross-linked at 120 000 μJ $cm^{-2}$ by using an ultraviolet Stratalinker 1800 (Stratagene, La Jolla). The blots were prehybridized for 3–4 h at 65˚C in rapid hybridization buffer (Amersham), and the membranes were hybridized overnight at 65˚C with the cDNA probe for VEGF or glyceraldehyde 3-phosphate dehydrogenase (GAPDH) (see below). The probed nylon membranes were then washed and exposed to radiographic film (GIBCO BRL Life Technologies Inc, Grand Island).

A human VEGF-specific 204-bp cDNA probe was a gift from Dr Brygida Berse (Harvard Medical School, Boston), and a GAPDH probe was purchased from the American Type Culture Collection (Manassas). The VEGF probe identifies all of the alternatively spliced forms of VEGF mRNA transcripts. Probes were purified by agarose gel electrophoresis by using the QIAEX Gel Extraction kit (QIAGEN Inc, Chatworth). Each cDNA probe was radiolabeled with (α-32P) deoxyribonucleotide triphosphate by using the random-priming technique with the Rediprime labeling system (Amersham).

### VEGF promoter-reporter activity in response to serum starvation

The effect of EGCG on the transcriptional regulation of VEGF by serum starvation of HT29 cells was examined by using transient transfection with a VEGF promoter (luciferase)-reporter construct. Full-length VEGF promoter cDNA, kindly provided by J Abraham (Scios Nova Inc, Mountain View), was subcloned into pGL3 by using standard techniques (Akagi et al, 1998). The following plasmids were used: pGL3-VEGF (containing the human VEGF promoter linked to the firefly luciferase reporter gene) (Promega, Madison), pRLTK (an internal control plasmid containing the herpes simplex thymidine kinase promoter linked to a constitutively active Renilla luciferase reporter gene), and pGL3 (plasmid vector alone as a negative control). HT29 cells (0.5–1.0 × 106) were seeded in 6-well plates, and the pRLTK and pGL3-VEGF constructs were cotransfected into cells with the FuGENETM6 Transfection Reagent (Boehringer Mannheim, Indianapolis) as described by the manufacturer. pRLTK and pGL3 were cotransfected as a negative control.

After cells were incubated in the transfection medium for 24 h, the medium was changed to standard medium and the cells were incubated for another 24 h, after which they were incubated in serum-free medium for 24 h. To determine whether EGCG could inhibit the increase in VEGF-promoter activity associated with serum deprivation, cells were treated with EGCG 1 h before being exposed to the serum-free condition. Cells were harvested with passive lysis buffer (Dual-Luciferase Reporter Assay System, Promega), and luciferase activity was determined with a single-sample luminometer, as outlined in the manufacturer’s protocol.

### Tumour cell inoculation and EGCG treatment

Six-week-old male athymic BALB/c nude mice were obtained from the National Cancer Institute’s Animal Production Area (Frederick) and were acclimated for 1 week. Mice were then injected subcutaneously with 106 viable HT29 cells. Beginning 2 days later, mice were given daily intraperitoneal injections of 1.5 mg EGCG or EC (control). (Our previous studies have shown no difference between mice injected with EC and those injected

© 2001 Cancer Research Campaign 

with PBS (data not shown), so we elected to use EC as the control condition.) Animals were observed daily for tumour growth, and when tumours appeared they were measured every 3 days. Tumour volume was calculated as $0.5 \times \text{length} \times \text{width}^2$ (length > width). All animal studies were conducted in accord with institutional guidelines approved by the Animal Care and Use Committee of The University of Texas MD Anderson Cancer Center.

### Necropsy and tissue preparation

Mice were killed by cervical dislocation 22 days after tumour-cell implantation. The tumours were excised, weighed, and sectioned and the tumour sections were either embedded in OCT compound and frozen at –70˚C or fixed in formalin.

### Immunohistochemical analysis

Tumour vessel formation was assessed immunohistochemically by staining the tumour sections for CD31 and proliferating cell nuclear antigen (PCNA) as follows. Formalin-fixed or paraffin-embedded sections were treated by standard deparaffinization, and sections frozen in OCT were treated by fixation in acetone and chloroform; immunohistochemical analyses then were performed as described elsewhere (Shaheen et al, 1999). Briefly, endogenous peroxidases were blocked with 3% $H_2O_2$ in methanol, slides were washed in PBS, incubated for 20 min with protein-blocking solution (PBS supplemented with 1% normal goat serum and 5% normal horse serum), incubated overnight at 4˚C with primary antibodies directed against CD31 or PCNA. Then the slides were washed again, incubated with protein-blocking solution, incubated for 1 h at room temperature with peroxidase-conjugated secondary antibodies, washed, incubated with DAB, washed, counterstained with haematoxylin, washed, mounted with Universal Mount, and dried on a 56˚C hot-plate. Negative controls were prepared with the same procedure but without the primary antibody.

### Immunofluorescent staining for CD31 and the TUNEL assay

Frozen tumour sections were stained by immunofluorescence for CD31 according to the above protocol with the following modifications. After sections were incubated overnight at 4˚C with the primary antibody, washed, and incubated with protein-blocking solution, they were incubated for 1 h at room temperature with a secondary antibody that was conjugated to Texas Red (red fluorescence), washed, and then TUNEL staining was performed according to the manufacturer's protocol. Briefly, the sections were fixed with 4% methanol-free paraformaldehyde, washed, permeabilized with 0.2% Triton X-100, washed, incubated with the kit's equilibration buffer, incubated with a reaction mix containing equilibration buffer, nucleotide mix, and the TdT enzyme at 37˚C for 1 h, incubated for 15 min at room temperature with 2 × standard saline citrate to stop the TdT reaction, washed, stained with DAPI mount (to visualize the nuclei), and glass coverslips were applied.

### Quantification of CD31, PCNA and TUNEL

Tumour vessels and PCNA-positive cells were evaluated by light microcopy, counted in five random 0.159-mm$^2$ fields at 100 × magnification, imaged digitally, and processed with Optimas Image Analysis software (Biscan, Edmond). We also quantified apoptosis with immunofluorescence by imaging sections digitally and processing them with Adobe Photoshop software (Adobe Systems, Mountain View). CD31-positive endothelial cells were detected by localized red fluorescence using a rhodamine filter. Tumour and endothelial cell apoptosis was visualized by localized green fluorescence using a fluorescein filter. Nuclei were detected by blue fluorescence of the DAPI with its respective filter. Cell counts were obtained in five random 0.011-mm$^2$ fields per slide at 400 × magnification. The percentage of apoptotic cells was determined as (number of apoptotic cells / total number of cells) × 100).

### Statistical analysis

Statistical comparisons among groups were made with the Mann–Whitney U-test for non-parametric data or Student's *t*-test for parametric data (promoter studies) (InStat Statistical Software, San Diego) at the 95% confidence level ($P < 0.05$ was considered statistically significant).

### RESULTS

### Erk-1 and -2 activation

Erk-1 and Erk-2 were found to be activated (phosphorylated) in serum-deprived HT29 cells, as previously reported (Jung et al, 1999), whereas the total levels of Erk-1 and Erk-2 did not change significantly. Our previous studies demonstrated that pretreating the cells with 1 mg ml$^{-1}$ of green tea extract inhibited the Erk-1 and -2 activation that had been induced by serum deprivation (data not shown). Purified EGCG, at 30 μM, markedly inhibited Erk-1 and -2 activation, but EGC, ECG, and EC at this concentration had no effect (Figure 1A). Next, we treated cells with 0, 10, 30, or 50 μM EGCG under serum-free conditions and found that EGCG inhibited the Erk-1 and -2 activation in serum-deprived HT29 cells in a dose-dependent manner (Figure 1B).

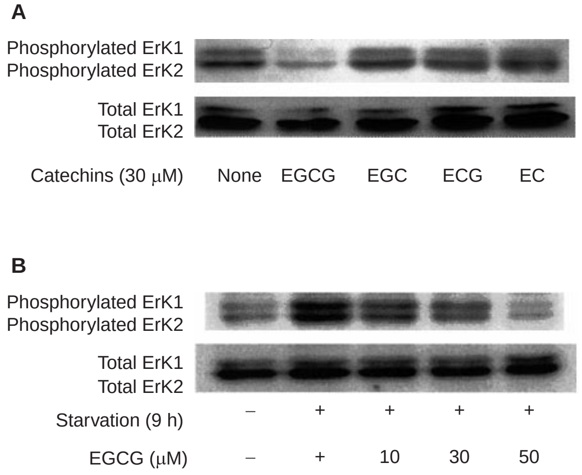

**Figure 1** EGCG, but not other tea catechins, blocks Erk-1 and -2 activation induced by serum deprivation. HT29 cells were treated with the indicated amounts of (-)-epigallocatechin gallate (EGCG), (-)-epigallocatechin (EGC), (-)-epicatechin gallate (ECG), or (-)-epicatechin (EC) in serum-free media for 9 h, and the cell lysates were analysed for phosphorylated and total forms of Erk-1 and -2 protein by Western blotting. Only EGCG blocked Erk-1 and -2 activity induced by serum starvation (**A**), and it did so in a dose-dependent fashion (**B**)

*© 2001 Cancer Research Campaign*

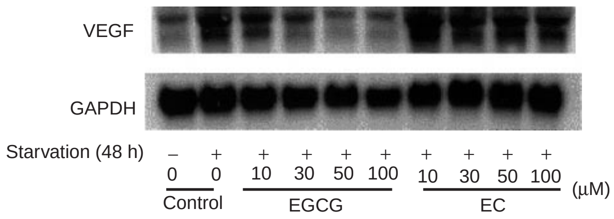

**Figure 2** EGCG inhibits the VEGF expression induced by serum deprivation. HT29 cells were treated with the indicated amounts of (-)-epigallocatechin gallate (EGCG) or (-)-epicatechin (EC) in serum-free medium for 48 h. Total RNA was isolated and analysed for VEGF and GAPDH mRNA by Northern blotting. EGCG inhibited VEGF expression in a dose-dependent fashion.

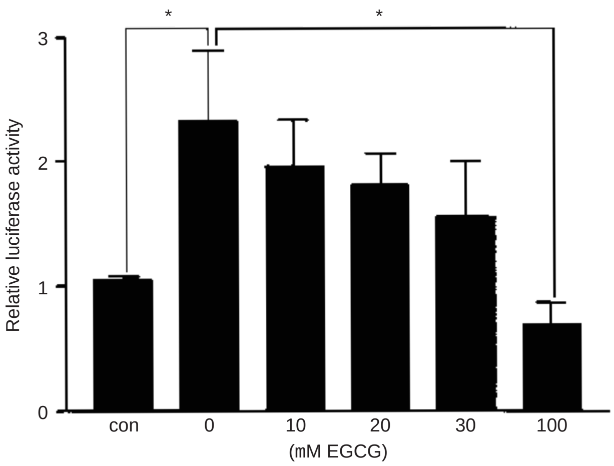

**Figure 3** EGCG inhibits the induction of VEGF promoter activity in colon carcinoma cells. HT29 cells were co-transfected with pGL3-VEGF (a VEGF promoter–luciferase-reporter construct) and pRLTK (control for transfection efficiency); co-transfection of pGL3 and pRLTK was used as a negative control. After 23 h, (-)-epigallocatechin gallate (EGCG) was added, and 1 h thereafter the medium was changed to serum-free medium. Cells were harvested after 24 h of serum starvation, protein was extracted, and luciferase activity was determined. Five separate experiments were done and results were standardized to reporter activity at $t = 0$ (contro; control values were set at 1 or 1.1 to allow for statistic analysis utilizing our computer software program). Bars indicate standard error of the mean.*$P < 0.05$ (unpaired Student's *T*-test)

### VEGF expression

Since Erk-1 and -2 activation is known to be involved in the induction of VEGF by serum deprivation, we next examined the effect of EGCG on VEGF expression. EGCG inhibited VEGF expression in serum-deprived HT29 cells in a dose-dependent manner, but EC did not affect VEGF expression at any dose (Figure 2). Neither EGC nor ECG (at 50 μM concentrations) affected VEGF expression in serum-starved cells (data not shown).

### Transcriptional regulation of VEGF

To examine the effect of EGCG on the transcriptional regulation of VEGF induced by serum starvation, we transiently transfected promoter-reporter constructs into HT29 cells. Cells transfected with pGL3-VEGF (promoter-reporter construct) and pRLTK (internal control) demonstrated an increase in VEGF promoter activity secondary to serum starvation. Treating cells with EGCG inhibited the activity of the VEGF promoter in a dose-dependent fashion (Figure 3).

### Growth of HT29 cells in vivo

Daily injections of EGCG (1.5 mg $mouse^{-1}$ $day^{-1}$) produced no signs of toxicity in athymic mice and effectively suppressed the growth of HT29 cells that had been implanted subcutaneously into those mice. At 22 days after tumour-cell implantation, tumour volume was inhibited by 61% (Figure 4A) and tumour weight was inhibited by 58% (Figure 4B) in the EGCG-treated group. In preliminary studies, we demonstrated that there was no difference in tumour size between mice injected with EC and those injected with PBS (data not shown); therefore we used EC as the control agent.

### Tumour angiogenesis and tumour cell proliferation

We used immunohistochemical staining for CD31 to reveal vessel formation in tumour sections. At 22 days after tumour-cell implantation, daily EGCG treatment had decreased the number of tumour vessels by 30% compared with that of controls

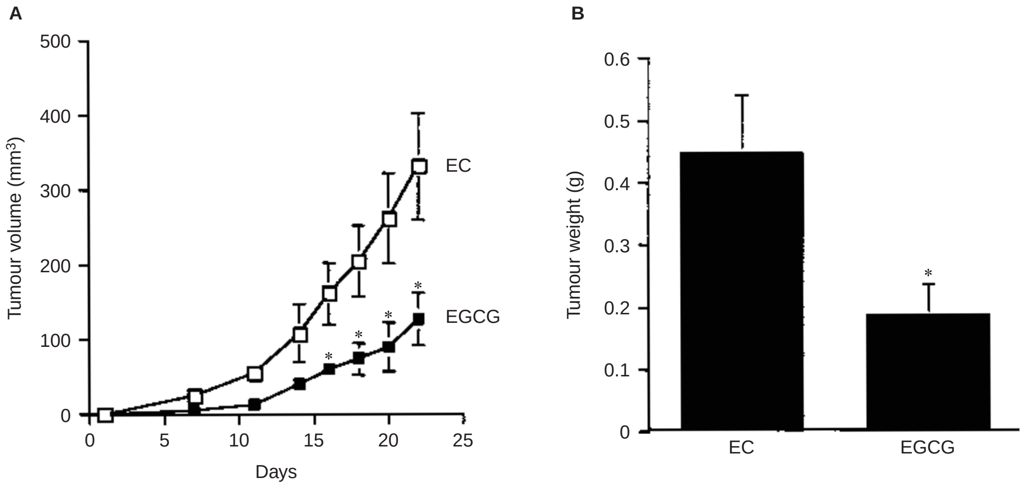

**Figure 4** EGCG reduces tumour volume and weight. At 22 days after tumour-cell implanatation, (-)-epigallocatechin gallate (EGCG) had inhibited tumour volume by 61% (**A**) and tumour weight by 58% (**B**) relative to tumours from mice treated with (-)-epicatechin (EC). Bars indicate standard error.*$P < 0.05$ (10 mice per group)

© 2001 Cancer Research Campaign

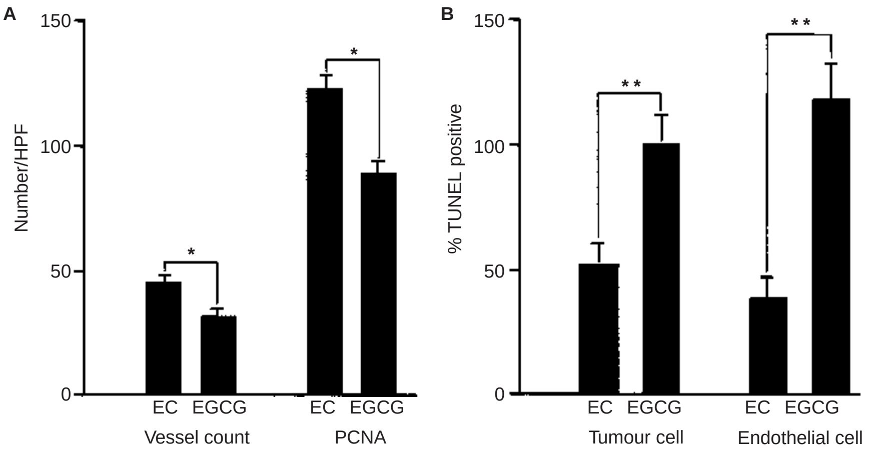

**Figure 5** EGCG inhibits tumour vascularity and tumour cell proliferation. Immunohistochemical staining of tumour sections for CD31 and proliferating cell nuclear antigen (PCNA) was used to quantify tumour vessels and tumour cell proliferation. **A** (-)-epigallocatechin gallate (EGCG) inhibited tumour vascularity by 30% and tumour cell proliferation by 27%. **B** Immunofluorescent double staining of tumour sections for CD31 and TdT-mediated dUTP nick-end labelling (TUNEL) was performed to quantify the percentages of tumour and endothelial cells undergoing apoptosis. EGCG treatment significantly increased apoptosis of both cell types over that of controls. HPF = high-power field. Bars indicate standard error.**P* < 0.05, ***P* < 0.001 (10 mice per group)

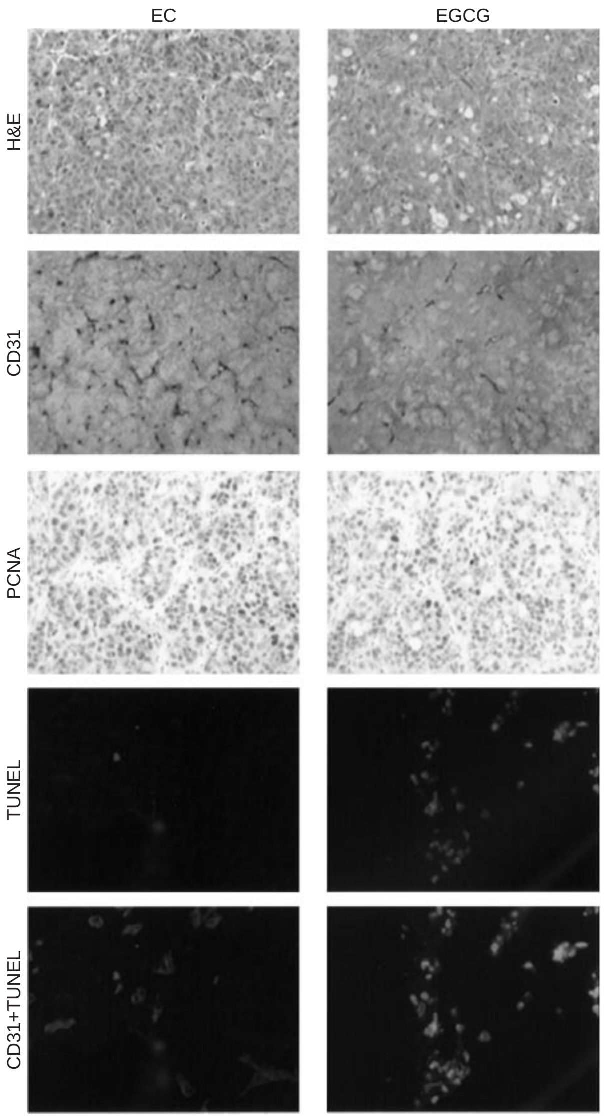

**Figure 6** Appearance of human colon cancer xenograft sections in nude mice. Tumour sections were stained with haematoxylin and eosin (row 1 = 40× magnification), immunohistochemically for CD31 (row 2 = 100×) and PCNA (row 3 = 100×); and immunofluorescently for TUNEL (row 4 = 400×) and sequential CD31 (red) and TUNEL (green) (row 5 = 400×). (-)-epigallocatechin gallate (EGCG) treatment led to decreases in number of tumour vessels (row 2) and tumour cell proliferation (row 3) and an increase in endothelial cell apoptosis (row 5) relative to treatment with (-)-epicatechin (EC) (column 1)

(Figure 5A). Tumour cell proliferation was evaluated by immunohistochemical staining for PCNA. Tumours from mice treated with EGCG had significantly less tumour cell proliferation (27%) than that of controls (Figure 5A).

### Apoptosis of tumour and endothelial cells

Immunofluorescent TUNEL staining with concurrent staining for CD31 was performed to quantify endothelial apoptosis in tumour sections (Shaheen et al, 1999). EGCG treatment produced a 1.9-fold increase in tumour cell apoptosis and a 3-fold increase in endothelial cell apoptosis, compared with those of controls (Figure 5B). The differences in microvessel density, tumour cell proliferation and endothelial cell apoptosis between the EC and EGCG groups are illustrated in Figure 6.

### DISCUSSION

EGCG is the most abundant of the green tea polyphenols, accounting for more than 40% of the total polyphenolic mixture (Stoner and Mukhtar, 1995). Several molecular mechanisms have been suggested for EGCG's observed anticancer effect, including suppression of ligand binding to the EGF receptor (Liang et al, 1997); inhibition of urokinase (Jankun et al, 1997), protein kinase C (Kitano et al, 1997), lipoxygenase, and cyclooxygenase activities (Stoner and Mukhtar, 1995); and induction of apoptotic cell death and arrest of the cell cycle (Ahmad et al, 1997; Fujiki et al, 1998) in tumour cells.

In the present study, we found that EGCG inhibited angiogenesis by blocking Erk-1 and Erk-2 activation and VEGF expression.

© 2001 Cancer Research Campaign

The Erk-1 and Erk-2 pathway is also thought to be essential in cellular growth and differentiation. Blockade of the Mek-Erk pathway suppresses growth of colon tumours in vivo (Sebolt-Leopold et al, 1999). Recently, Erk-1 and Erk-2 has been reported to be an important signalling cascade that leads to overexpression of VEGF mRNA (Jung et al, 1999; Milanini et al, 1998). We previously showed that VEGF is up-regulated in serum-starved HT29 cells through the activation of Erk-1 and Erk-2 (Jung et al, 1999). Milanini et al (1998) also found that maximal transcriptional activation of VEGF in fibroblasts was Erk-dependent. The EGCG-mediated inhibition of Erk-1 and -2 activation may be an early cellular event that is partly responsible for the anticancer effect of EGCG.

The exact mechanism by which ECGC inhibits the activation of Erk-1 and -2 in serum-starved cells is not known. One possible explanation is that EGCG could inhibit kinases that are involved in Erk-1 and -2 activation. EGCG is known to be a strong metal ion chelator (Yang and Wang, 1993). Since some receptor kinases depend on divalent cations for their activity (Mahadevan et al, 1995), EGCG could inhibit the activity of receptor kinases by chelating the divalent cations.

VEGF is a potent and unique angiogenic protein that has specific mitogenic and chemotactic effects on vascular endothelial cells. Studies from our laboratory and others suggest that VEGF is the angiogenic factor that is most closely associated with induction and maintenance of the neovasculature in human colon cancer (Warren et al, 1995; Ellis et al, 1996; Takahashi et al, 1996). Our present results show that treatment of mice with EGCG resulted in marked inhibition of the growth, vascularity, and proliferation of human tumour xenografts in nude mice. We also found that EGCG induced significant endothelial cell apoptosis, a result that supports our earlier contention that VEGF is an in vivo survival factor for tumour endothelium (Shaheen et al, 1999).

These findings suggest that down-regulation of VEGF by EGCG may lead to endothelial cell apoptosis within tumours, which could not only inhibit new blood vessel formation and tumour growth but also could lead to tumour cell apoptosis. These findings are supported by other recent reports that green tea could inhibit tumour growth by suppressing blood vessel growth (Cao and Cao, 1999; Swiercz et al, 1999).

These studies have demonstrated that green tea, and more specifically EGCG, can inhibit tumour growth in vivo, possibly by inhibiting the formation of new blood vessels. These findings may partially explain the antineoplastic effects associated with drinking green tea. A complete knowledge of the molecular mechanism or mechanisms involved with the anti-tumour efficacy of green tea polyphenols may be useful in devising better strategies for cancer therapy.

### ACKNOWLEDGEMENTS

This work was supported by research funds from the Chonnam University Research Institute of Medical Sciences (CURIMS 98-B-130) (YDJ), Korea Research Fund from the Ministry of Education (BAS), the University Cancer Foundation (YDJ, LME), and the Gillson Longenbaugh Foundation (GEG, LME). The authors thank Christine Wogan and Joan Small for editorial assistance.

### REFERENCES

Ahmad N, Feyes DK, Nieminen AL, Agarwal R and Mukhtar H (1997) Green tea constituent epigallocatechin-3-gallate and induction of apoptosis and cell cycle arrest in human carcinoma cells. *J Natl Cancer Inst* **89**: 1881–1886

Ahn HY, Hadizadeh KR, Seul C, Yun YP, Vetter H and Sachinidis A (1999) Epigallocathechin-3 gallate selectively inhibits the PDGF-BB-induced intracellular signaling transduction pathway in vascular smooth muscle cells and inhibits transformation of sis-transfected NIH 3T3 fibroblasts and human glioblastoma cells (A172). *Mol Biol Cell* **10**: 1093–1104

Akagi Y, Liu W, Zebrowski B, Xie K and Ellis LM (1998) Regulation of vascular endothelial growth factor expression in human colon cancer by insulin-like growth factor-I. *Cancer Res* **58**: 4008–4014

Asano Y, Okamura S, Ogo T, Eto T, Otsuka T and Niho Y (1997) Effect of (-)-epigallocatechin gallate on leukemic blast cells from patients with acute myeloblastic leukemia. *Life Sci* **60**: 135–142

Cao Y and Cao R (1999) Angiogenesis inhibited by drinking tea [letter]. *Nature* **398**: 381

Cobb MH and Goldsmith EJ (1995) How MAP kinases are regulated. *J Biol Chem* **270**: 14843–14846

Davis RJ (1993) The mitogen-activated protein kinase signal transduction pathway. *J Biol Chem* **268**: 14553–14556

Ellis LM, Liu W and Wilson M (1996) Down-regulation of vascular endothelial growth factor in human colon carcinoma cell lines by antisense transfection decreases endothelial cell proliferation. Surger, **120**: 871–878

Ellis LM, Staley CA, Liu W, Fleming RY, Parikh NU, Bucana CD and Gallick GE (1998) Down-regulation of vascular endothelial growth factor in a human colon carcinoma cell line transfected with an antisense expression vector specific for c-src. *J Biol Chem* **273**: 1052–1057.

Folkman J (1995) Angiogenesis in cancer, vascular, rheumatoid and other disease. *Nature Med* **1**: 27–31

Fujiki H, Suganuma M, Okabe S, Sueoka N, Komori A, Sueoka E, Kozu T, Tada Y, Suga K, Imai K and Nakachi K (1998) Cancer inhibition by green tea. *Mutat Res* **402**: 307–310

Hibasami H, Komiya T, Achiwa Y, Ohnishi K, Kojima T, Nakanishi K, Akashi K and Hara Y (1998) Induction of apoptosis in human stomach cancer cells by green tea catechins. *Oncol Rep*, **5**: 527–529

Jankun J, Selman SH, Swiercz R and Skrzypczak-Jankun E (1997) Why drinking green tea could prevent cancer [see comments]. *Nature* **387**: 561

Ji BT, Chow WH, Hsing AW, McLaughlin JK, Dai Q, Gao YT, Blot WJ and Fraumeni JF Jr (1997) Green tea consumption and the risk of pancreatic and colorectal cancers. *Int J Cancer* **70**: 255–258

Jung YD, Nakano K, Liu W, Gallick GE and Ellis LM (1999) Extracellular signal-regulated kinase activation is required for up-regulation of vascular endothelial growth factor by serum starvation in human colon carcinoma cells. *Cancer Res* **59**: 4804–4807

Kitano K, Nam KY, Kimura S, Fujiki H and Imanishi Y (1997) Sealing effects of (-)-epigallocatechin gallate on protein kinase C and protein phosphatase 2A. *Biophys Chem* **65**: 157–164

Liang YC, Lin-shiau SY, Chen CF and Lin JK (1997) Suppression of extracellular signals and cell proliferation through EGF receptor binding by (-)-epigallocatechin gallate in human A431 epidermoid carcinoma cells. *J Cell Biochem* **67**: 55–65

Mahadevan D, Thanki N, Aroca P, McPhie P, Yu JC, Beeler J, Santos E, Wlodawer A and Heidaran MA (1995) A divalent metal ion binding site in the kinase insert domain of the alpha-platelet-derived growth factor receptor regulates its association with SH2 domains. *Biochemistry* **34**: 2095–2106

Milanini J, Vinals F, Pouyssegur J and Pages G (1998) p42/p44 MAP kinase module plays a key role in the transcriptional regulation of the vascular endothelial growth factor gene in fibroblasts. *J Biol Chem* **273**: 18165–18172

Paschka AG, Butler R and Young CY (1998) Induction of apoptosis in prostate cancer cell lines by the green tea component, (-)-epigallocatechin-3-gallate. *Cancer Lett* **130**: 1–7

Rogers AE, Hafer LJ, Iskander YS and Yang S (1998) Black tea and mammary gland carcinogenesis by 7,12-dimethylbenz[a]anthracene in rats fed control or high fat diets. *Carcinogenesis* **19**: 1269–1273

Sebolt-Leopold JS, Dudley DT, Herrera R, Van Becelaere K, Wiland A, Gowan RC, Tecle H, Barrett SD, Bridges A, Przybranowski S, Leopold WR and Saltiel AR (1999) Blockade of the MAP kinase pathway suppresses growth of colon tumors in vivo. *Nat med* **5**: 810–816

Shaheen RM, Davis DW, Liu W, Zebrowski BK, Wilson MR, Bucana CD, McConkey DJ, McMahon G and Ellis LM (1999) Antiangiogenic therapy

© 2001 Cancer Research Campaign targeting the tyrosine kinase receptor for vascular endothelial growth factor receptor inhibits the growth of colon cancer liver metastasis and induces tumour and endothelial cell apoptosis. *Cancer Res* **59**: 5412–5416
Stoner GD and Mukhtar H (1995) Polyphenols as cancer chemopreventive agents. *J Cell Biochem Suppl* **22**: 169–180
Swiercz R, Skrzypczak-Jankun E, Merrell MM, Selman SH and Jankun J (1999) Angiostatic activity of synthetic inhibitors of urokinase type plasminogen activator. *Oncol Rep* **6**: 523–526
Takahashi Y, Kitadai Y, Bucana CD, Cleary KR and Ellis LM (1995). Expression of vascular endothelial growth factor and its receptor, KDR, correlates with vascularity, metastasis, and proliferation of human colon cancer. *Cancer Res* **55**: 3964–3968
Takahashi Y, Bucana CD, Liu W, Yoneda J, Kitadai Y, Clearly KR and Ellis LM (1996) Platelet-derived endothelial cell growth factor in human colon cancer angiogenesis: role of infiltrating cells [see comments]. *J Natl Cancer Inst* **88**: 1146–1151
Takahashi Y, Tucker SL, Kitadai Y, Koura AN, Bucana CD, Cleary KR and Ellis LM (1997) Vessel counts and expression of vascular endothelial growth factor as prognostic factors in node-negative colon cancer. *Arch Surg* **132**: 541–546
Warren RS, Yuan H, Matli MR, Gillett NA and Ferrara N (1995) Regulation by vascular endothelial growth factor of human colon cancer tumorigenesis in a mouse model of experimental liver metastasis. *J Clin Invest* **95**: 1789–1797
Yang CS and Wang ZY (1993) Tea and cancer. *J Natl Cancer Inst* **85**: 1038–1049
Yu GP, Hsieh CC, Wang LY, Yu SZ, Li XL and Jin TH (1995) Green-tea consumption and risk of stomach cancer: a population-based case-control study in Shanghai, China. *Cancer Causes Control* **6**: 532–538

 © 2001 Cancer Research Campaign
